# Viability of ex-vivo myography as a diagnostic tool for rectus abdominis muscle electrical activity collected at Cesarean section within a diamater cohort study

**DOI:** 10.1186/s12938-022-01042-2

**Published:** 2022-10-14

**Authors:** David R. A. Reyes, Angelica M. P. Barbosa, Floriano F. Juliana, Quiroz B. C. V. Sofia, Sarah M. B. Costa, Raghavendra L. S. Hallur, Eusebio M. A. Enriquez, Rafael G. Oliveira, Patricia de Souza Rossignolli, Cristiane Rodrigues Pedroni, Fernanda C. B. Alves, Gabriela A. Garcia, Joelcio F. Abbade, Carolina N. F. Carvalho, Luis Sobrevia, Marilza V. C. Rudge, Iracema I. M. P. Calderon, The Diamater Study Group, F. P. Souza, T. Lehana, C. F. O. Graeff, C. G. Magalhães, R. A. A. Costa, S. A. M. Lima, M. R. K. Rodrigues, S. L. Felisbino, W. F. Barbosa, F. J. Campos, G. Bossolan, J. E. Corrente, H. R. C. Nunes, P. S. Rossignoli, Á. N. Atallah, Z. I. Jármy-Di Bella, S. M. M. Uchôa, M. A. H. Duarte, E. A. Mareco, M. E. Sakalem, N. M. Martinho, D. G. Bussaneli, M. I. G. Orlandi, C. Pascon, T. D. Dangió, F. Piculo, G. M. Prata, R. E. Avramidis, A. B. M. Magyori, G. T. A. Nava, T. C. D. Caldeirão, R. H. L. Shetty, J. P. C. Marcondes, M. L. S. Takemoto, C. B. Prudencio, F. A. Pinheiro, C. I. Sartorao Filho, S. B. C. V. Quiroz, T. Pascon, S. K. Nunes, B. B. Catinelli, F. V. D. S. Reis, M. O. Menezes, N. J. Santos, L. Takano, A. M. Carr, L. F. Iamundo, H. C. M. Bassin, V. P. Barbosa, M. Jacomin, A. J. B. Silva, I. O. Lourenço, J. Marosticadesá, I. P. Caruso, L. T. Rasmussen, V. K. C. Nogueira, J. T. Ribeiro-Paes, D. C. H. França, H. V. M. Bastos, M. L. A. Heliodoro, M. N. Kuroda, H. L. Carvalho

**Affiliations:** 1 Department of Gynecology and Obstetrics, Botucatu Medical School (FMB), São Paulo State University (UNESP), Botucatu, São Paulo CEP18618-687 Brazil; 2 Department of Physiotherapy and Occupational Therapy, School of Philosophy and Sciences, São Paulo State University (UNESP), Marilia, Brazil; 3 Centre for Biotechnology, Pravara Institute of Medical Sciences (Deemed to Be University), Loni-413736, Rahata Taluk, Ahmednagar District, Ahmednagar, Maharashtra India; 4 Cellular and Molecular Physiology Laboratory (CMPL), Department of Obstetrics, Division of Obstetrics and Gynaecology, School of Medicine, Faculty of Medicine, Pontificia Universidad Católica de Chile, 8330024 Santiago, Chile; 5 Department of Physiology, Faculty of Pharmacy, Universidad de Sevilla, 41012 Seville, Spain; 6 University of Queensland Centre for Clinical Research (UQCCR), Faculty of Medicine and Biomedical Sciences, University of Queensland, Herston, QLD 4029 Australia; 7 Department of Pathology and Medical Biology, Division of Pathology, University of Groningen, University Medical Center Groningen (UMCG), Groningen, The Netherlands

**Keywords:** Contractility, Rectus abdominis muscle, C-section, Myography, Skeletal muscle

## Abstract

**Background:**

*Ex-vivo* myography enables the assessment of muscle electrical activity response. This study explored the viability of determining the physiological responses in muscles without tendon, as rectus abdominis muscle (RAM), through *ex-vivo* myography to assess its potential as a diagnostic tool.

**Results:**

All tested RAM samples (five different samples) show patterns of electrical activity. A positive response was observed in 100% of the programmed stimulation. RAM 3 showed greater weight (0.47 g), length (1.66 cm), and width (0.77 cm) compared to RAM 1, RAM 2, RAM 4 and RAM 5 with more sustained electrical activity over time, a higher percentage of fatigue was analyzed at half the time of the electrical activity. The order of electrical activity (Mn) was RAM 3 > RAM 5 > RAM 1 > RAM 4 > RAM 2. No electrical activity was recorded in the Sham group.

**Conclusions:**

This study shows that it is feasible to assess the physiological responses of striated muscle without tendon as RAM, obtained at C-section, under *ex vivo* myography. These results could be recorded, properly analyzed, and demonstrated its potential as a diagnostic tool for rectus abdominis muscle electrical activity.

## Background

The Rectus Abdominis Muscle (RAM), a striated muscle without tendon, extending along the abdomen to the pubic symphysis, undergoes physiological adaptations during pregnancy [1–5]. As a striated muscle, the main focus of research has been on contractile muscle cells [6]. RAM has synergistic contractile activity with the pelvic floor muscles in labor and urinary continence mechanisms [5, 7, 8]. The importance of the RAM analysis may be underscored by the fact that some complications during the pregnancy, such as gestational diabetes mellitus (GDM), cause serious morphological changes [9–11] leading to atrophy and, as a consequence, urinary incontinence (UI) up 2 years after C-section [1, 9, 12–14].

The challenge of identifying such abnormalities of RAM contractility is overcome by its accessibility during C-sections allowing detailed studies of the muscle contractility [15–17]. For this analysis, the *ex-vivo* myography of RAM samples may clarify the knowledge of the pathophysiology of diseases and disorders that affect the RAM [17–22]. Although it is widely known that the *ex-vivo* myography is used to record muscle contractility through the myogram, a tracing graphic recording [18, 19, 23], that requires tendon attachments through the myograph [24–26]. Currently, there is no information available on the *ex-vivo* electrical activity in skeletal muscle, such as RAM without tendon. Therefore, efforts are needed to be made for the success of the RAM analysis through myography that will serve as an important tool for female muscle functional evaluation, its disorders and the pathophysiological mechanisms involved [26, 27].

The main goal of this study was to determine the viability of detecting electrical activity through *ex-vivo* myography in RAM samples without tendon collected during C-section as a physiological response to assess its potential as a diagnostic tool, for continuous electric stimulation, the fatigue and loss of tissue viability.

## Results

Baseline maternal demographics, RAM sample characteristics and the programmed parameters of *ex-vivo* myography are summarized in Table 1. Of the five patients with RAM samples collected, all are from term pregnancy, three are primiparous, one has obesity plus chronic hypertension and pre-eclampsia and all are continent and normoglycemic.

**Table 1:** Baseline maternal characteristics of ex vivo myograph—programmed parameters and RAM activity

Experimental group							Sham group
Clinical characteristics of pregnant woman							
Age	In years	24	27	25	31	24	
Parity		Primiparous	Secondpregnancy	Primiparous	Primiparous	Secondpregnancy	
Gestational week	In weeks	38.2	38.4	38.6	37	38.3	
Obesity	Yes (Y) or No (N)	N	Y	N	N	N	
Chronic arterial hypertension	Yes (Y) or No (N)	N	Y	N	N	N	
Preeclampsia	Yes (Y) or No (N)	N	Y	N	N	N	
Pregnancy specific urinary incontinence	Yes (Y) or No (N)	N	N	N	N	N	
Gestational diabetes mellitus	Yes (Y) or No (N)	N	N	N	N	N	

RAM activity	Description	Response					Krebs solution and elastic ribbon
		RAM 1	RAM2	RAM 3	RAM 4	RAM 5	
Total number of major peak	Major peaks/5 min	248	248	248	248	248	–
Total number minor peak	Minorpeaks/5 min	248	248	248	248	248	–
Response rate	Compared to previously programmed (%)	100.0	100.0	100.0	100.0	100.0	–
Contraction force (mN)		8.50 ± 0.67	6.67 ± 5.03	17.5 ± 12.5	6.7 ± 0.4	15.9 ± 17.6	–
Characteristics RAM samples							
Weight (grams)	0.30 ± 0.09						
Width (cm)	0.51 ± 0.09						
Length (cm)	1.50 ± 0.19						

**Fig. 1:**
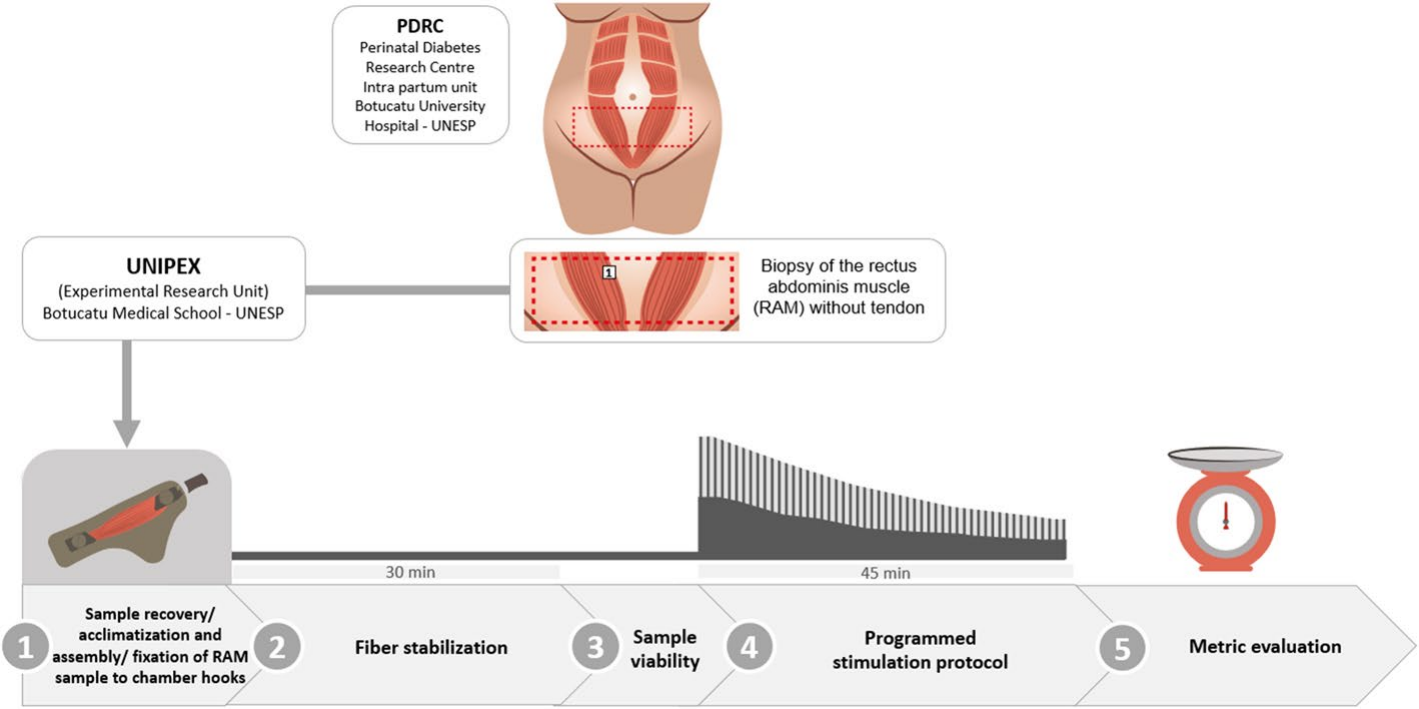
Four steps of the experiment performed from the RAM without tendon

**Fig. 2:**
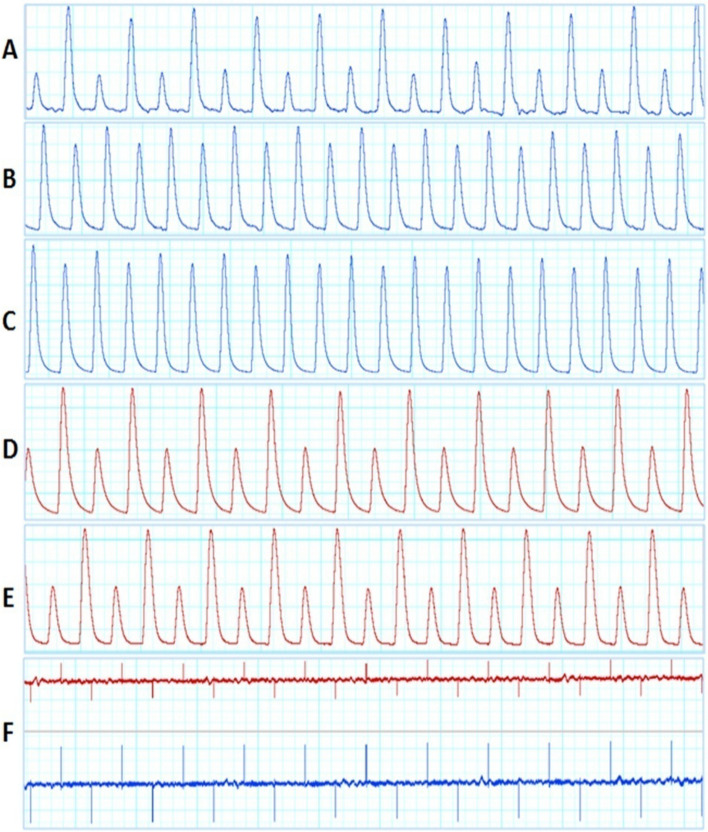
Response to the programmed electrical stimulation in five RAM samples. The myograph shows the behavior of elastic ribbon (sham) under the same electrical stimulation as RAMs; **A**–**E** represents RAM 1–5 and **F** represents the sham group

A100% of RAM samples without tendons responded positively to programmed electrical stimulus allowing to record and to analyze the muscle functionality (Fig. 1, Table 1). All these 5 RAM samples presented a serial peak of contractile activity with two different patterns, viz, three samples (RAM 1, 4, and 5) showed contractility activity characterized by high and low peaks consecutively, and two samples (RAM 2 and 3) showed contractility activity with only by high peaks (Fig. 2A–E, Table 1). Both record patterns decreased in force at half (22.5 min) of the experiments and all samples remained viable during 1 h and 15 min experiments. The elastic ribbon (inert material) from the sham group showed no activity, as expected (see Fig. 2F). The response to the programmed electrical stimulation in the five RAM samples was measured considering the same major and minor peak values, and 100% of response in the *ex-vivo* myography. RAM 3 showed higher parameters of weight, width and length, average peak heights greater than 17.5 ± 12.5 compared to the other RAM samples, initial peak greater than 20.5 mN. However, a higher percentage of fatigue or loss of viability in RAM 3 was observed with a decrease in muscle contraction strength at 22.5 min compared with the other samples (Figs. 2Cand 3).

**Fig. 3:**
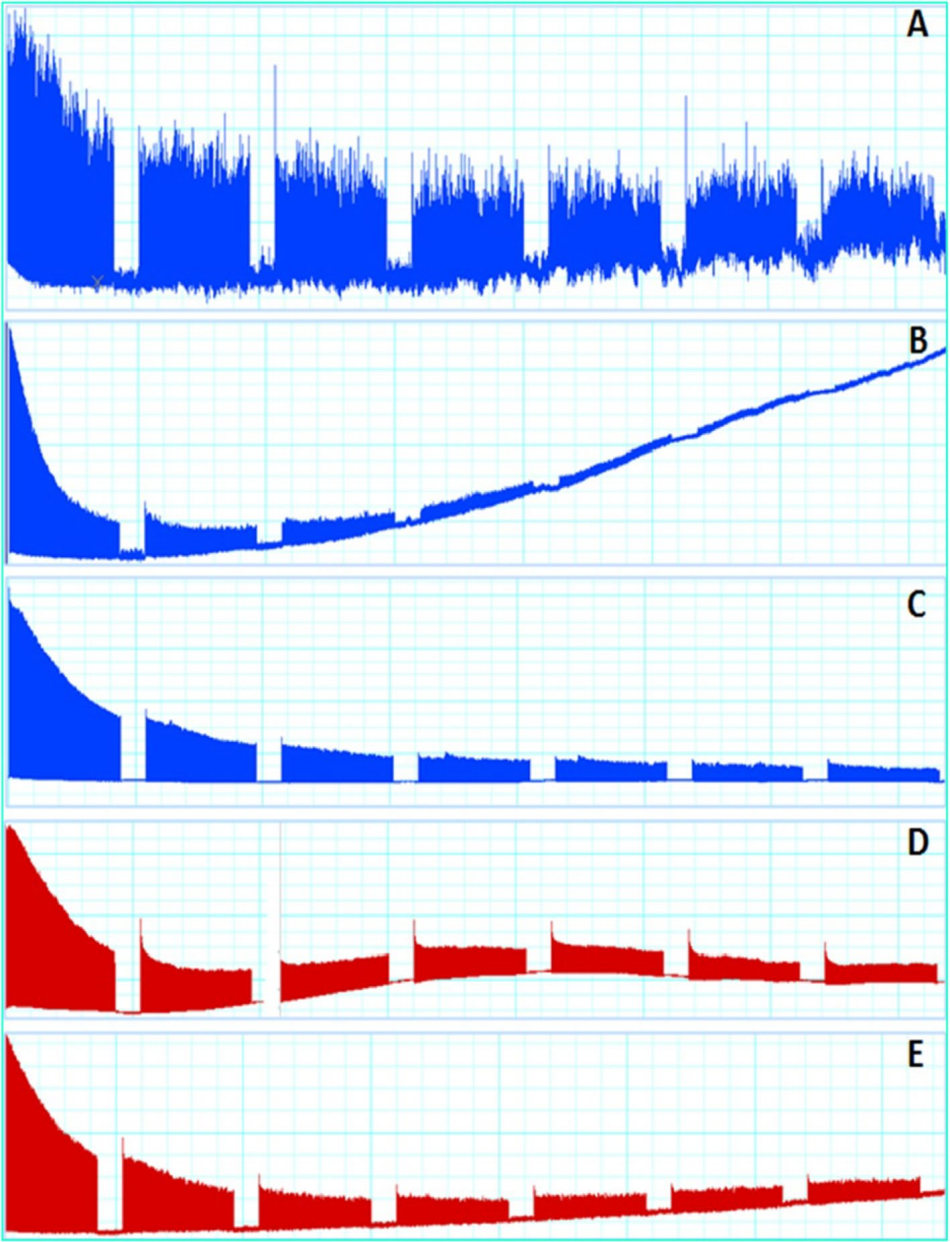
Fatigue andloss of the viability of five RAM. **A**–**E** Samples at half the time of the electrical activity

RAM 2 showed lower average peak heights and had an initial peak height of 10.8 mN and a lower peak of fatigue or loss of viability at 22.5 min (see Figs. 2, 3 and Table 1).

Figure 3 shows muscle fatigue or loss of viability of five RAM (RAM 1–5) samples, respectively. RAM 1 (6.40 mN), RAM 2 (1.56 mN), RAM 3 (8.23mN), RAM 4 (7.23mN), RAM 5 (4.42mN) which is at half the time of the electrical activity.

## Discussion

This study shows that fresh striated muscle without tendon reacted to electrical stimuli and stays viable showing a 100% response to a programmed electrical stimulation. This experimental model provides information on the performance and functionality of striated muscle without tendons from pregnant women, opening a promising field that would allow making inferences, analyses, evaluations, and measurements, using their tissues through the ex vivo myography method in humans.

It is reported that women with GDM showed altered muscle morphology and 3D ultrasound which may influence the functionality of RAM with consequences for UI [14, 28]. We are aware of other modalities for studying contractility, such as electromyography, ultrasound, morphology, etc. However, myography may be considered an approach to detect the contractile activity in muscles without tendon not only in UI due to GDM but also to allow testing of the effects of *ex-vivo* drug application to human and animal tissues. The electrical stimulation and other parameters used in this study were based on previous literature on different muscles with tendons [24, 25]. This study shows that RAM reacted to electrical stimulation with contraction force and fatigue. These results may, however, be affected by parameters that may interfere with the contraction force of RAM samples, such as sample size, width, and length, and the region of the RAM collection, which varies from superficial, medial, and deep RAM [29], the presence of fat, fascia, the muscular collagen concentration, and the presence of slow and fast fibers percentage.

The electrical activity of RAM biopsies after 11.8 Hz electrical stimulation (force ~ 10 mN)was lower than other striated muscles, such as the extensor digitorum muscle longus (EDL) which showed a force ~ 80 mN [24] and the gastrocnemius muscle with a force ~ 62 mN [30–32]. Thus, the absence of a tendon may have a great impact on the lower observed contraction force in RAM. As verified in RAM 2, a lower contraction force may be linked to the concentration of collagen which is largely found in striated muscles during pregnancy [10, 14]. RAM 3 showed a higher initial peak, greater weight, width, and length, probably related to a greater number of motor units and sarcomeres recruited during muscle contraction likely capable of performing a stronger contraction. Since RAM3 was collected before birth its larger force may relate to less muscle fiber damage compared to RAM 2 collected after birth which presented a lower initial peak, weight, and length [31–34].

A selection of best possible parameters to describe the RAM *ex-vivo* myography technique may include: (i) the two types of contraction,(ii) the peak contraction to verify contraction strength and relaxation time, (iii) the loss of strength characterizing muscle fatigue, and (iv) loss of muscle viability by having less processing time from collection to experiment that can be graphically manifested by a rising trend of the waves, showing loss of sustainability of contractility of the muscle fiber. It is critical to consider the region of the RAM sample collection, which varies from superficial, medial and deep RAM [29]. It is known that RAM regions act in series [34] and the differences in the force of contraction can be verified by evaluating its electrical activity by separated. In addition, the absence of a tendon may have a great impact on the lower observed contraction force.

The functionality of the RAM to the electrical stimulus is characterized by a larger peak and a smaller peak. This response may be related to the response of the total number of slow and fast fibers captured by the myograph transducer. Previous studies confirmed that the composition and amount of fiber types slow and fast are key factors in muscles’ functionality [35]. The limitations of this study are the reduced number of samples analyzed, and the collection procedure and characteristics of the RAM biopsies obtained through an invasive surgical procedure. RAM may vary in size, weight, length, fiber composition, and the presence of fasciae.

## Conclusions

This pilot study demonstrates the viability of *ex-vivo* myography in striated muscles without tendons. The results obtained in this study are the first description of the RAM response under electrical stimulation, such as contraction force and fatigue, supporting the potential of RAM biopsy obtained at C-section as a tissue that can be used in *ex-vivo* myography. This experimental model provides information on the performance and functionality of striated muscle without tendons in pregnant women. Myography is an approach that might be relevant in detecting the contractile activity in muscles without tendon not only in UI due to GDM but also to allow testing of the effects of *ex-vivo* drug application on human and animal tissues. The *ex-vivo* myography of fresh RAM samples is an approach suggested to be included in the full analysis of hyperglycemic myopathy as an underlying mechanism of long-term UI due to GDM.

## Materials and methods

### Recruitment

The pregnant women were recruited at the Perinatal Diabetes Research Center (PDRC) of Botucatu Medical School-UNESP, Brazil, between 24 and 28th weeks of gestation. The objectives and importance of the research were explained to the pregnant woman. Written informed consent to include in the study was obtained. The RAM samples were collected during C-section.

### Study population and groups

This viability study constitutes part of a Diamater cohort study protocol [13]. This current study followed the CONSORT-2010 statement for the extension to randomized pilot and viability trials [36]. This study was approved by the Institutional Research Bureau of Botucatu Medical School-UNESP (protocol nº CAAE: 82225617.0.0000.5411). Three primiparous and two second-birth pregnant women over 18 years of age were included. The recruitment was carried out from August 2019 to March 2021. Two groups were used in this study, the study group (RAM biopsy) and the sham group (elastic ribbon).

### RAM samples collection

The RAM biopsy collection was performed by the medical staff after surgical suture of the myometrium, visceral and parietal peritoneum during C-section with medical and or obstetric.

indication, within a maximum period of 10 min after fetal extraction. In non-emergency C-sections, RAM samples could be obtained before childbirth. Immediately after resection, a RAM sample of 1 cm was collected, the fat and connective tissue were removed, and the muscle samples were placed in a Falcon tube containing 5 mL Krebs solution (118.5 μM NaCl, 30 μM KCl, 290 μM NaHCO_3_, 9 μM MgSO_4_, 9 μM KH_2_PO_4_, 20 μg CaCl_2_, 5.5 g D-glucose, 300 μM l-arginine, pH 7.4) and kept at 4 °C until analysis. The time between sample collection and the beginning of ex*-vivo* myography was a maximum of 30 min.

### Equipment

This study used a DMT-myograph system (model 820MS DMT-Danish Myo Technology^®^, Ann Arbor, Michigan USA)to assess the electrical activity. The chamber contains hooks, where the muscles are mounted to the edges, one end of the chamber, contains a force transducer that converts kinetic energy (muscle responses, under electrical stimulation) into an electrical signal that can be recorded and analyzed with PowerLab equipment (PowerLab Data Acquisition System, AD Instruments, São Paulo, Brazil) and LabChart software (LabChart 8 for Windows, AD Instruments). The equipment was calibrated according to the manufacturer’s recommendations and as per the training obtained at the DTM Company (Ann Arbor, Michigan, USA).

### Muscle mounting method

The settings adjusted in the stimulation equipment were performed based on previous literature [24, 25, 37] and performed using other muscles with tendons and tissues [24, 38, 39].

#### Sample recovery/acclimatization and assembly/fixation of RAM sample to chamber hooks (1.^st^ step)

An isolated organ bath was performed in Krebs solution with carbogenic gas for 15 min, previously heated to 37 °C, to recover the mechanical properties of the muscle. The RAM fragment was mounted vertically, following the arrangement of the fibers, in the myograph chamber filled with 4 mL of Krebs solution with continuous gas flow and minimal bubbling, which provides essential gases to maintain muscle in conditions that simulate the organic environment.

#### Fiber stabilization (2^nd^ step)

the Krebs solution was changed twice at 15 min intervals, with a fixed tension force of 10 mN applied at each Krebs change. To carry out the 3rd and 4th stages, electrical stimulation was performed using platinum electrodes, placed parallel to the longitudinal axis, at 0.8 cm from the muscle tissue and integrated into the myographic cells, which provide electrical stimuli through the electrodes, by a series of predefined quadratic waves, due to its characteristic digital pulse (0–1). These conditions allow the observation of minimum and maximum points (dual pulse wave, gradient, ramp, sine, and triangle), controlled by MyoD analog output software (Danish Myo Technology^®^, Michigan, USA), with voltage-controlled (20 V) computer software and constant current management. To capture muscle responses with greater sensitivity and precision, muscle stimulation was performed with stimulation equipment (Grass Model S48, Danish Myo Technology^®^, Michigan, USA);

#### Sample viability (3^rd^ step)

to verify the viability, electrical stimulation was performed with three stimuli of biphasic wave force of 10 mN, the voltage of 20 V, and pulse width of 25 mS. Approximate run time of 2 min.

#### Programmed stimulation protocol (4^th^ step)

the application of the continuous alternating current stimulation protocol was performed with a fixed pulse train. Pulse voltage (volts): 20 V, pulse width (milliseconds): 25 ms, pulse interval (milliseconds): 60 ms, power: 10mN, pulse frequency (Hertz): 11.8 Hz, pause between pulse trains (milliseconds): 500 ms, pulse trains in the group: 500, repeat group: 10 times, pause between groups of trains (milliseconds): 60000 ms, total run time: 45 min.

#### Metric evaluation (5^th^ step)

After the stimulation, the equipment was turned off and the parameters of the muscle sample, length(cm), width(cm) and weight(g) were determined. In the analysis of the results, the average of three measurements performed to normalize the results was considered. A digital caliper (STARFER^®^, Vargem Grande do Sul, SP, Brazil) was used to measure muscle length and a digital analytical balance (model SHIMADZU^®^ ATX224K ern ABT220-4NM, Kyoto, Japan) was used to weigh the fragment. Muscle weight was recorded in grams (dry weight).

### Analysis of muscle biomechanics response under electrical stimulation of RAM

#### Frequency per quadrant

A quadrant with a standard time interval was used for all analyses. A 10:1 speed was taken and the interval time was 5 min. All the highest and lowest peaks are counted in this 5 min time interval. These results corroborated the contraction frequency in relation to the electrical stimulus used to confirm whether the muscle contracted or not according to the programmed training (electrical stimulus performed by the equipment as set up in the software).

#### Peak height and time to peak

The speed was decreased between the biggest and the smallest peaks so they became more evident. The highest and lowest peak height values were recorded and the strength and response to the stimulus (muscle function) were calculated.

#### Force

The speed was increased to detect the descent of the graph (decreased strength which probably characterizes fatigue or viability loss).

#### Datapad

The values of the highest and lowest peaks were extracted from the datapad, this way we were able to assess when the peak started to be lowest (decrease in strength and fatigue).

#### Pulse calculation

According to the equipment set up there are 2 repetitions (two stimuli) in 1 s (2 stimuli/second), each repetition has 1 ms of duration separated by 500 ms which is the interval time named “rest”. Finally, the overall electrical pulses were calculated, it would be 2 stimuli per second multiplied by the period of time analyzed.

### Statistical analysis

This is a descriptive study to document the viability of the *ex-vivo* myograph technique in RAM samples of pregnant women. Therefore, there were no comparisons, and basic statistical analyzes of mean, median, and standard deviation were applied to properly assess the data for each sample.

## Data Availability

Contact the corresponding author for data requests.

## Abbreviations

RAM: Rectus abdominis muscle
C-section: Cesarean section
UI: Urinary incontinence
GDM: Gestational diabetes mellitus
EDL: Digitorum muscle longus
PDRC: Perinatal diabetes research center
DMT: Danish Myo technology
cm: Centimeters
g: Grams

## References

[1] Vesentini G, Barbosa AMP, Damasceno DC, Marini G, Piculo F, Matheus SMM (2020). Alterations in the structural characteristics of rectus abdominis muscles caused by diabetes and pregnancy: a comparative study of the rat model and women. PLoS ONE.

[2] Piculo F, Marini G, Vesentini G, Morceli G, Damasceno DC, Sobrevia L (2020). Pregnancy-specific urinary incontinence in women with gestational hyperglycaemia worsens the occurrence and severity of urinary incontinence and quality of life over the first year postpartum. Eur J Obstet Gynecol Reprod Biol.

[3] Biviá-Roig G, Lisón JF, Sánchez-Zuriaga D (2018). Changes in trunk posture and muscle responses in standing during pregnancy and postpartum. PLoS ONE.

[4] Idoate F, Calbet JAL, Izquierdo M, Sanchis-Moysi J (2011). Soccer attenuates the asymmetry of rectus abdominis muscle observed in non-athletes. PLoS ONE.

[5] Amorim AC, Cacciari LP, Passaro AC, Silveira SRB, Amorim CF, Loss JF (2017). Effect of combined actions of hip adduction/abduction on the force generation and maintenance of pelvic floor muscles in healthy women. PLoS ONE.

[6] Csapo R, Gumpenberger M, Wessner B (2020). Skeletal muscle extracellular matrix—what do we know about its composition, regulation, and physiological roles?A narrative review. Front Physiol.

[7] Madill SJ, McLean L (2006). Relationship between abdominal and pelvic floor muscle activation and intravaginal pressure during pelvic floor muscle contractions in healthy continent women. Neurourol Urodyn.

[8] Barbosa AMP, Enriquez EMA, Rodrigues MRK, Prudencio CB, Atallah ÁN, Reyes DRA (2020). Effectiveness of the pelvic floor muscle training on muscular dysfunction and pregnancy specific urinary incontinence in pregnant women with gestational diabetes mellitus: a systematic review protocol. PLoS ONE.

[9] Vesentini G, Marini G, Piculo F, Damasceno DC, Matheus SMM, Felisbino SL (2018). Morphological changes in rat rectus abdominis muscle induced by diabetes and pregnancy. Brazilian J Med Biol Res.

[10] Marini G, Piculo F, Vesentini G, Damasceno DC, Delella FK, Calderon IMP (2018). The influence of hyperglycemia on the remodeling of urethral connective tissue in pregnant rats. Eur J Obstet Gynecol Reprod Biol.

[11] Piculo F, Marini G, Barbosa AMP, Damasceno DC, Matheus SMM, Felisbino SL (2014). Urethral striated muscle and extracellular matrix morphological characteristics among mildly diabetic pregnant rats: translational approach. Int Urogynecol J.

[12] Barbosa AMP, Adriano Dias GM, Calderon IMP, Witkin S, RudgeI MVC (2011). Urinary incontinence and vaginal squeeze pressure two years post-cesarean delivery in primiparous women with previous gestational diabetes mellitus. Clinics.

[13] Rudge MVC, Souza FP, Abbade JF, Hallur RLS, Marcondes JPC, Piculo F (2020). Study protocol to investigate biomolecular muscle profile as predictors of long-term urinary incontinence in women with gestational diabetes mellitus. BMC Pregnancy Childbirth.

[14] Vesentini G, Barbosa AMP, Floriano JF, Felisbino SL, Costa SMB, Piculo F (2020). Deleterious effects of gestational diabetes mellitus on the characteristics of the rectus abdominis muscle associated with pregnancy-specific urinary incontinence. Diabetes Res Clin Pract.

[15] Dimassi K, Halouani A, Kammoun A, Ami O, Simon B, Velemir L (2021). The extraperitoneal French AmbUlatory cesarean section technique leads to improved pain scores and a faster maternal autonomy compared with the intraperitoneal Misgav Ladach technique: a prospective randomized controlled trial. PLoS ONE.

[16] Dodd JM, Anderson ER, Gates SGR (2014). Surgical techniques involving the uterus at caesarean section. Cochrane Database Syst Rev.

[17] Wakahara T, Shiraogawa A (2019). Effects of neuromuscular electrical stimulation training on muscle size in collegiate track and field athletes. PLoS ONE.

[18] Gluppe SL, Hilde G, Tennfjord MK, Engh ME, Bø K (2018). Effect of a postpartum training program on the prevalence of diastasis recti abdominis in postpartum primiparous women: a randomized controlled trial. Phys Ther.

[19] Shamsi M, Sarrafzadeh J, Jamshidi A, Zarabi V, Pourahmadi MR (2016). The effect of core stability and general exercise on abdominal muscle thickness in non-specific chronic low back pain using ultrasound imaging. Physiother Theory Pract.

[20] Rahe-Meyer N, Pawlak M, Weilbach C, Osthaus WA, Ruhschulte H, Solomon C (2008). Complex myograph allows the examination of complex muscle contractions for the assessment of muscle force, shortening, velocity, and work in vivo. Biomed Eng Online.

[21] Rahe-Meyer N, Weilbach C, Karst M, Pawlak M, Ahmed A, Piepenbrock S (2007). In vivo myograph measurement of muscle contraction at optimal length. Biomed Eng Online.

[22] Rahe-Meyer N, Winterhalter M, Ahmed AI, Weilbach C, Gross M, Piepenbrock S (2007). Assessment of precision and reproducibility of a new myograph. Biomed Eng Online.

[23] Tarasova O, Sjöblom-Widfeldt N, Nilsson H (2003). Transmitter characteristics of cutaneous, renal and skeletal muscle small arteries in the rat. Acta Physiol Scand.

[24] Hakim CH, Wasala NB, Duan D (2013). Evaluation of muscle function of the extensor digitorum longus muscle *ex vivo* and tibialis anterior muscle in situ in mice. J Vis Exp.

[25] Oishi PE, Cholsiripunlert S, Gong W, Baker AJ, Bernstein HS (2010). Myo-mechanical analysis of isolated skeletal muscle. J Vis Exp.

[26] Xiao ZG, Menon C (2019). A review of force myography research and development. Sensors.

[27] Wu YT, Gomes MK, da Silva WH, Lazari PM, Fujiwara E (2020). Integrated optical fiber force myography sensor as pervasive predictor of hand postures. Biomed Eng Comput Biol.

[28] Nunes SK, Rudge CVC, Quiroz SCBV, Hallur RL, Prudencio CB, Pinheiro FA (2019). Impact of gestational diabetes mellitus on sexual function: a case–control study. J Women’s Heal.

[29] Brown SHM, Banuelos K, Ward SR, Lieber RL (2010). Architectural and morphological assessment of rat abdominal wall muscles: comparison for use as a human model. J Anat.

[30] Carosio S, Barberi L, Rizzuto E, Nicoletti C, Del PZ, Musarò A (2013). Generation of ex vivo-vascularized Muscle Engineered Tissue (X-MET). Sci Rep.

[31] Grange RW, Houston ME (1991). Simultaneous potentiation and fatigue in quadriceps after a 60-second maximal voluntary isometric contraction. J Appl Physiol.

[32] Tieland M, Trouwborst I, Clark BC (2018). Skeletal muscle performance and ageing. J Cachexia Sarcopenia Muscle.

[33] Lee JH, Jun HS (2019). Role of myokines in regulating skeletal muscle mass and function. Front Physiol.

[34] Rassier DE, MacIntosh BR (2002). Sarcomere length-dependence of activity-dependent twitch potentiation in mouse skeletal muscle. BMC Physiol.

[35] Blaauw B, Schiaffino S, Reggiani C, Terjung R (2013). Mechanisms modulating skeletal muscle phenotype. Comprehensive physiology.

[36] Eldridge SM, Chan CL, Campbell MJ, Bond CM, Hopewell S, Thabane L (2016). CONSORT 2010 statement: extension to randomised pilot and v trials. Pilot Feasibility Stud.

[37] Campos R, Justo AFO, Mónica FZ, Cogo JC, Moreno RA, de Souza VB (2018). Electrical field-induced contractions on crotalus durissus terrificus and bothrops jararaca aortae are caused by endothelium-derived catecholamine. PLoS ONE.

[38] del Campo L, Ferrer M, Andrés V, Dorado B (2015). Wire myography to study vascular tone and vascular structure of isolated mouse arteries BT—Methods in mouse atherosclerosis. Methods in mouse atherosclerosis methods in molecular biology.

[39] Lavie A, Shinar S, Hiersch L, Ashwal E, Yogev Y, Aviram A (2018). Uterine electrical activity, oxytocin and labor: translating electrical into mechanical. Arch Gynecol Obstet.

